# Causal Association of Adipose Tissue with Bladder Cancer and the Mediating Effects of Circulating Metabolites: A Mendelian Randomization Study

**DOI:** 10.7150/jca.100152

**Published:** 2024-10-21

**Authors:** Song Yang, Jun Jian, Xiaojie Zhao, Lei Wang, Zhiyuan Chen, Xiuheng Liu

**Affiliations:** Department of Urology, Renmin Hospital of Wuhan University, Wuhan, China; Institute of Urologic Disease, Renmin Hospital of Wuhan University, Wuhan, China.

**Keywords:** Adipose tissue, Bladder cancer, Circulating metabolites, Mendelian randomization

## Abstract

**Background:** Previous studies have indicated that there is an association between obesity and bladder cancer (BCa). However, the relationship between fat distribution, which is more representative of the risk of obesity, and BCa remains unclear. This study aimed to investigate the causal relationship between fat distribution and BCa, and the mediating role of circulating metabolites.

**Methods:** The necessary data were obtained from a large Genome-Wide Association Studies (GWAS) database. Two-sample and two-step Mendelian randomization (MR) analyses were performed to investigate the association between fat distribution and BCa, as well as the mediating effect of circulating metabolites. The inverse variance weighted (IVW) method was the main analysis method. Heterogeneity tests, horizontal pleiotropy analyses, Mendelian randomization pleiotropy residual sum and outlier (MR-PRESSO) tests, and leave-one-out analyses were performed to assess the stability of the results.

**Results:** The IVW method indicated that abdominal subcutaneous adipose tissue adjusted for body mass index (BMI) and height (ASATadj) and abdominal subcutaneous/gluteofemoral adipose tissue (ASAT/GFAT) increased the risk of BCa. The odds ratio (OR) for ASATadj was 1.78 (95% CI=1.27-2.50, p=0.001) and that for ASAT/GFAT was 1.64 (95% CI=1.01-2.66, p=0.047). Furthermore, two-step MR analysis revealed that the effect of ASAT/GFAT on BCa was mediated by valine (proportion mediated: 7.13%, 95% CI = 3.57%-10.69%, p=0.045).

**Conclusions:** Our research shows that, unlike most studies which focus on visceral fat, ASAT also impacts human health by increasing the risk of BCa, with the blood metabolite valine involved in this process. Monitoring and reducing ASAT accumulation can help reducce the disease burden of BCa.

## Introduction

Bladder cancer (BCa) is one of the most prevalent malignant tumors affecting the urinary system, with approximately 570,000 new cases and 210,000 deaths reported worldwide each year^1^. The occurrence and development of BCa is a complex pathological process, influenced by various factors. Smoking is the primary pathogenic factor for BCa, with approximately 50% of patients having a history of smoking^2^. Other factors such as obesity, exposure to carcinogens, and a high-fat diet may also contribute to the risk of developing BCa^3^, ^4^. Due to its high recurrence rate, BCa requires regular monitoring and follow-up, resulting in significant economic burden^5^. Further clarification of the factors involved in the occurrence and development of BCa is crucial for the development of early prevention and treatment strategies.

Obesity is a significant risk factor for numerous diseases, including cancer. It is estimated that overweight and obesity account for 8% of all cancers in high-income countries, and this burden is projected to increase further^6^. Obesity is defined as the excessive accumulation of adipose tissue throughout the body, and can be categorized into two main types: visceral adipose tissue (VAT) and subcutaneous adipose tissue (SAT)^7^. In contrast to SAT, VAT exhibits greater metabolic activity^8^. Research has indicated that abdominal fat measured by CT or MRI is more effective than traditional indicators such as body mass index (BMI) and waist circumference (WC) in assessing the metabolic risk associated with obesity^9^. However, most evidence regarding abdominal fat and disease risk comes from retrospective analyses and observational studies.

Mendelian randomization (MR) is an innovative epidemiological method that utilizes data from genome-wide association studies (GWASs) and employs single nucleotide polymorphisms (SNPs) as instrumental variables (IVs) to uncover causal relationships. MR addresses issues such as confounders and reverse causality, which are inherent limitations of observational studies, and provides a practical approach for conducting large-scale randomized controlled trials. In this study, we utilized data from a comprehensive GWAS database to investigate the potential association between fat distribution and bladder tumors, while also exploring the mediating role of circulating metabolites.

## Materials and methods

### Study design

**Figure 1:**
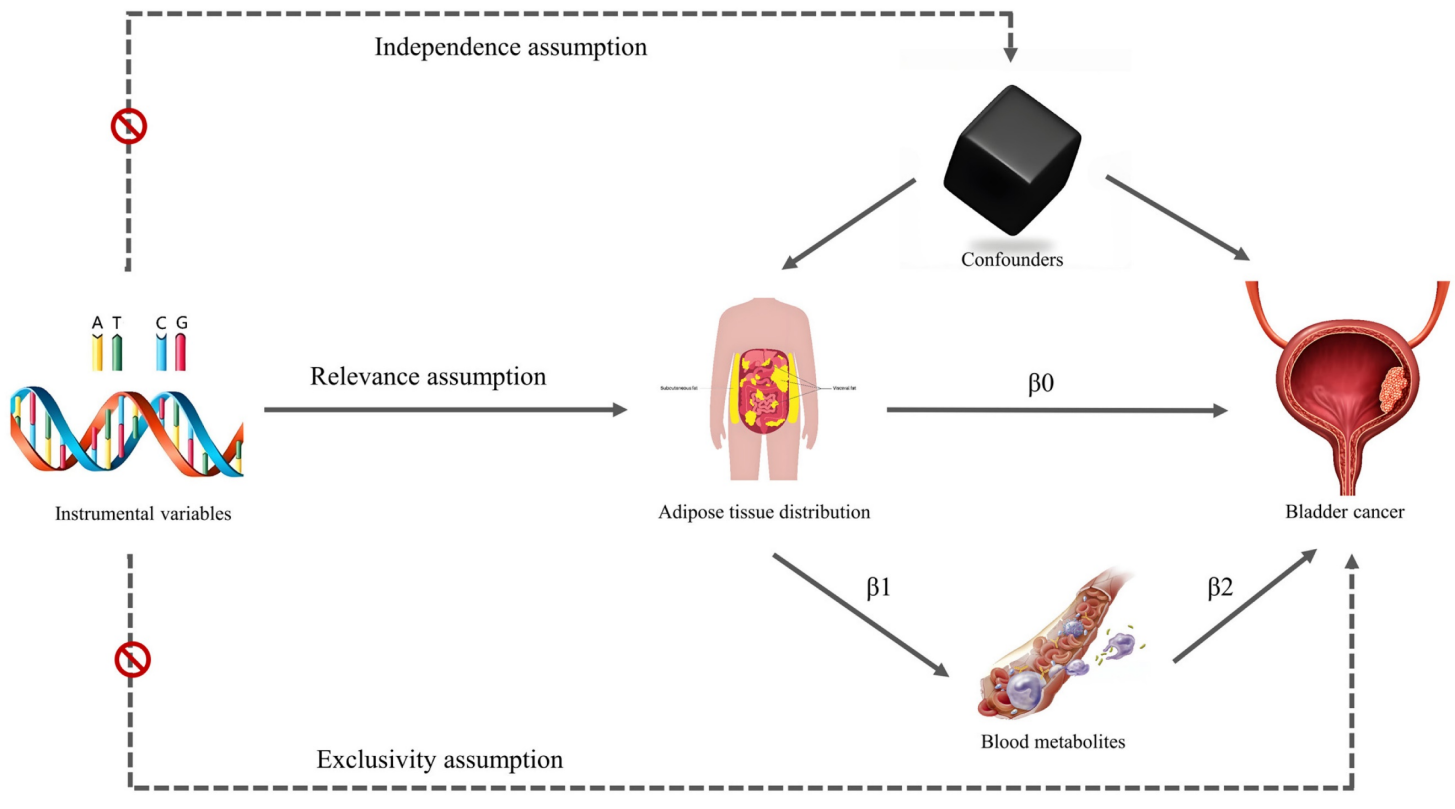
Conceptual framework for the study design.

We used MR and mediation analysis to explore and evaluate the relationship between fat distribution and bladder tumors, and whether this relationship was mediated by circulating metabolites. The overall design of this study is illustrated in Figure 1. The data utilized in the study were obtained from publicly available databases, therefore no approval from an institutional ethics review board was necessary.

### Data collection

This study primarily explores the causal relationship between fat located in different areas (such as visceral fat or subcutaneous fat) and BCa. Given that BMI affects fat distribution, we focus on using GWAS summary data that directly measures fat, with adjustments made for BMI, and ensuring these data include larger sample sizes. After a thorough search, summary data for fat distribution were obtained from the Cardiovascular Disease Knowledge Portal. The data were derived from MR images of 38,965 participants (51% female, 49% male) in the UK Biobank. VAT, abdominal subcutaneous (ASAT), and gluteofemoral (GFAT) adipose tissue volumes were measured. Additionally, the three indicators were adjusted for BMI and height (VATadj, ASATadj and GFATadj), and their ratios to each other were calculated (VAT/ASAT, VAT/GFAT and ASAT/GFAT).

Given that the population of exposure was from the UK Biobank, we opted to acquire GWAS summary data for bladder tumors from the FinnGen consortium. At the beginning of the study, data from the latest tenth version of the database were used. Our focus was on GWAS summary statistics for benign neoplasms of bladder (261 cases and 411920 controls), carcinoma *in situ* of the bladder (695 cases and 314185 controls), and malignant neoplasms of the bladder (2193 cases and 314193 controls).

For the GWAS summary data for circulating metabolites, we utilized the Nightingale Health data published in 2020. The study recruited a total of approximately 120,000 volunteers, aged between 37 and 73 years old, 54% of whom were women. EDTA plasma samples were collected approximately 4 hours after the last meal, and a total of 168 measurements of absolute levels and 81 measurements of ratios were performed^10^. The 168 indicators with absolute concentrations were used for MR analysis. Comprehensive data on these circulating metabolites can be accessed from the MRC-IEU GWAS database.

### Selection of instrumental variables (IVs)

The IVs in MR analysis should meet several conditions (Figure 1): 1. The IVs should be significantly associated with the exposure factor (relevance assumption); 2. No association between IVs and confounders (independence assumption); 3. The IVs weren't associated with outcome, but were only associated with outcome through exposure factors (exclusivity assumption). To select eligible IVs for the study, the following criteria were established: 1. SNPs related to exposure must meet a GWAS significance threshold of p < 5×10-8, and their association with exposure factors was evaluated using the F statistic (F = β^2^ / se^2^). Those with an F-statistic less than 10 were considered weak IVs and excluded from further analysis; 2. r2 < 0.001 with a clump distance of 10,000 kb was set to ensure independence from linkage disequilibrium; 3. The minor allele frequency threshold was set at 0.01 for outcome data extraction.

Lastly, LDtrait, an online tool for exploring phenotypic associations, was utilized for meticulous SNP analysis, particularly in relation to BCa and its risk factors such as smoking and Occupational carcinogen exposure, and so on 11, 12.

### Statistical analysis

#### Primary analysis

Several methods were used to explore the causal associations between fat distribution and circulating metabolites in bladder tumors, including inverse variance weighted (IVW), MR Egger regression and weighted median (WM). Among them, the results of IVW analysis are our focus, due to its ability to provide an unbiased estimate of causal effects in the absence of horizontal pleiotropy^13^. The MR results are presented as odds ratios (ORs) with corresponding 95% confidence intervals (95% CIs). Additionally, MR Egger and WM analysis methods were used as supplementary measures to IVW. The results were deemed reliable when the p-value of IVW method was less than 0.05 and the OR direction of MR Egger was aligned with that of IVW.

We further conducted a multivariate MR analysis of blood metabolites, which exhibit a significant causal relationship with BCa, to eliminate their potential interaction.

#### Mediation analysis

Fat distribution and circulating metabolites that showed significant associations with bladder tumors were included in the mediation analysis. A two-step MR design was used to analyse the total and indirect effects of fat distribution and circulating metabolites on bladder tumors. The total effect was denoted as β0, while the indirect effect was calculated as β1×β2 (Figure 1). The proportion of indirect effects was calculated by β1×β2/β0, and the delta method was used to calculate the 95% CI of the proportion of indirect effects.

#### Reverse MR analysis and Steiger directionality test

We performed reverse MR analysis of AT, circulating metabolites, and BCa to confirm that there was no inverse association among them. The remaining steps are analogous to the forward MR analysis described earlier. Additionally, we conducted a Steiger directionality test during the forward MR analysis to further validate the directionality from exposure to outcome.

#### Sensitivity analysis

We used Cochran's Q test to evaluate the heterogeneity of the analysis results. In cases of heterogeneity (p < 0.05), the Mendelian randomization pleiotropy residual sum and outlier (MR-PRESSO) method was used to identify abnormal SNPs and calculate the causal effect after removing outliers^14^. The "leave-one-out" method was applied to systematically remove single SNPs to evaluate the robustness of the results. Additionally, we used a funnel plot to examine the symmetry of the scatter distribution and further determine whether any outliers were present among the included SNPs in our analysis. Furthermore, we assessed horizontal pleiotropy using the intercept of MR-Egger regression analysis. Horizontal pleiotropy was indicated when there was a significant deviation from zero with p < 0.05 in the intercept term.

All analyses were conducted using the R Studio platform and R packages, including the TwoSampleMR (0.5.7) and forestploter (1.1.0) packages for statistical analysis and visualization.

## Results

### The selected IVs

After removing weak IVs and addressing linkage disequilibrium, we identified 165 SNPs associated with 9 fat distribution indicators (Table S1), characterized by F statistics ranging from 26.94 to 170.75, confirming the absence of weak IVs. Phenotypic association tests were conducted for each SNP using LDtrai, and three SNPs (rs2384054, rs1421085, rs62106258) directly associated with BCa or its risk factors were removed. Ultimately, 162 SNPs were included for further analysis.

### Total effect of fat distribution on bladder tumors

**Figure 2:**
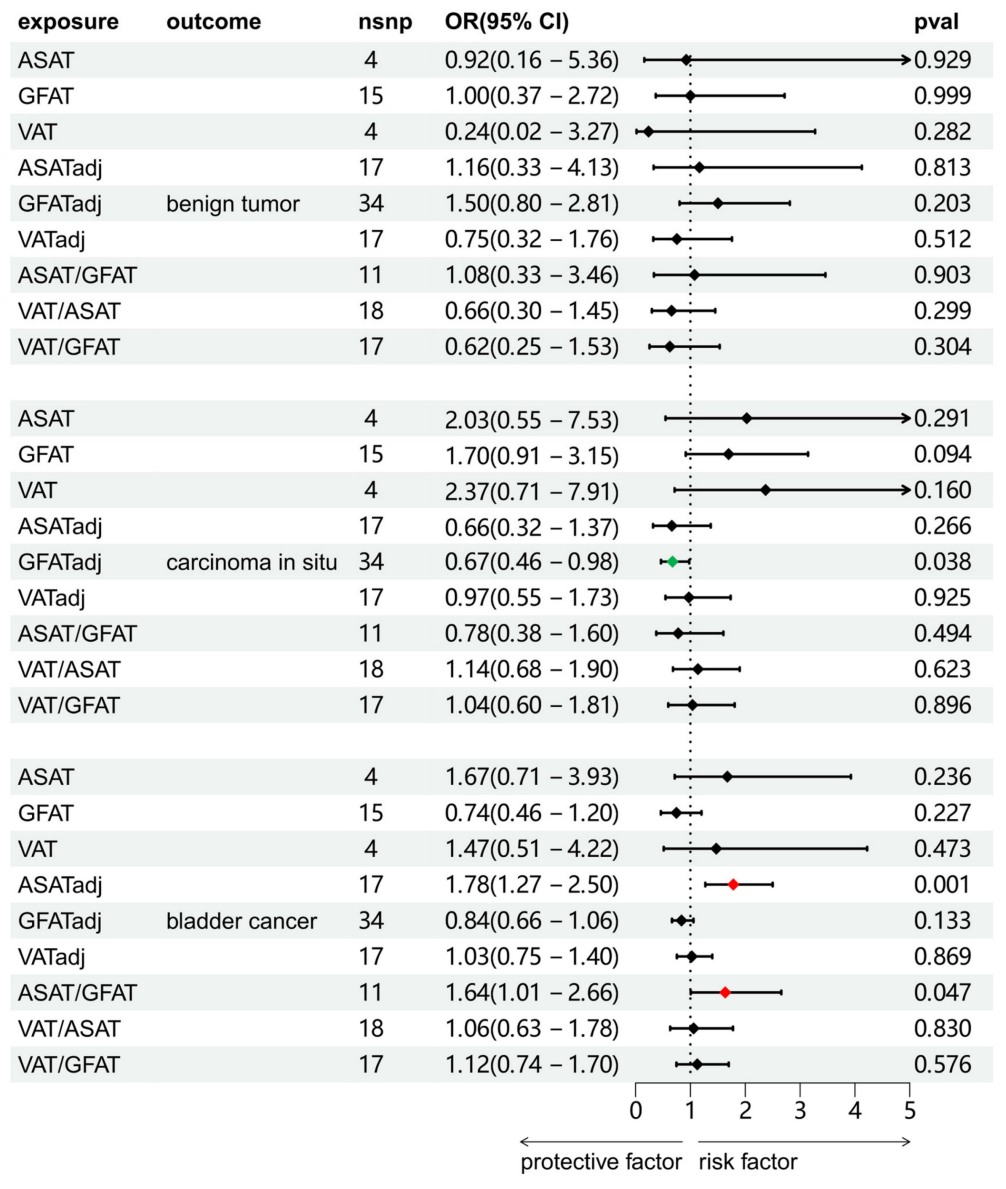
Two-sample MR analysis results of the causal relationship between fat distribution and bladder tumors. The results were from the IVW method.

**Figure 3:**
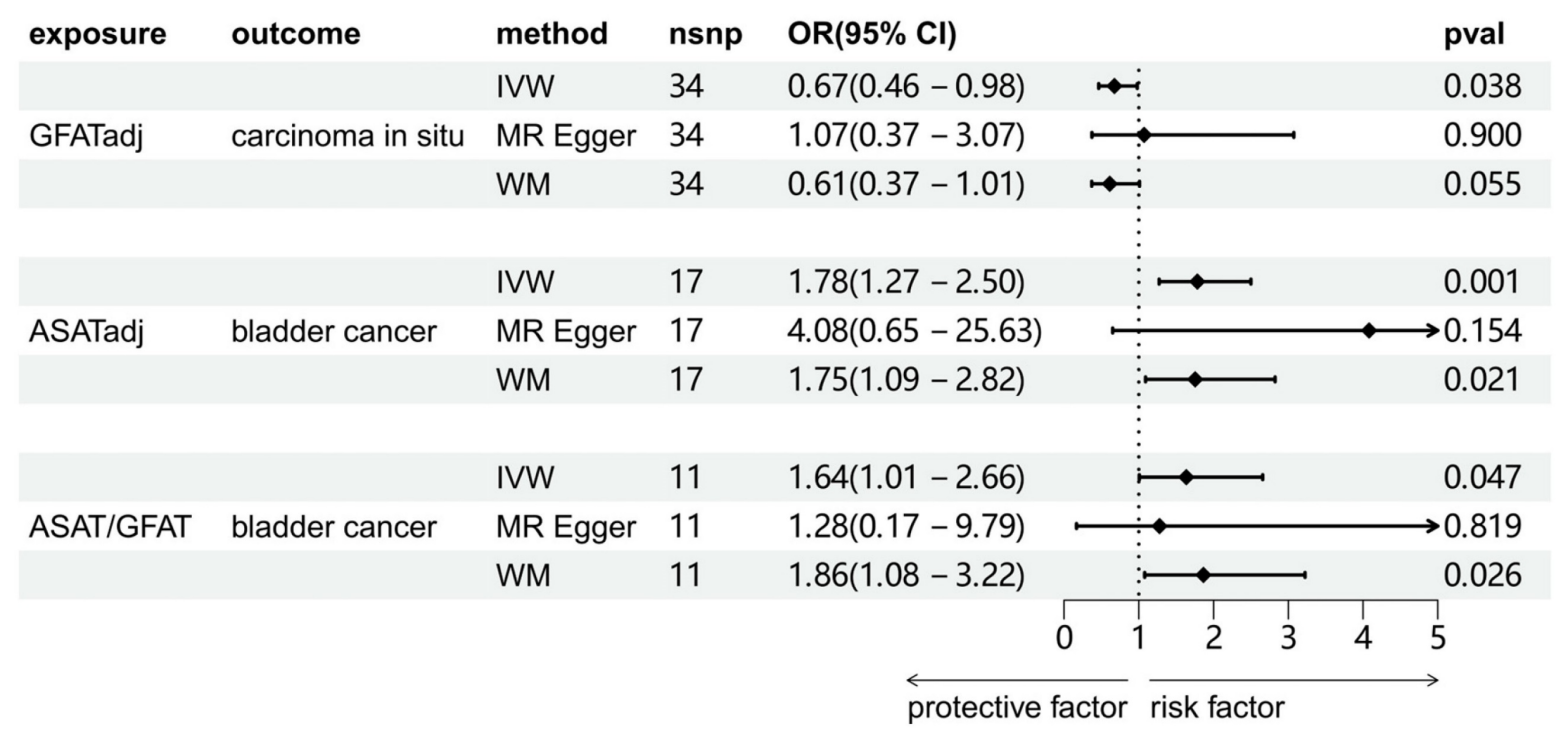
The results of three MR analysis methods of significant causality between adipose tissue and bladder tumors. IVW: inverse variance weighted method; MR Egger: MR Egger regression method; WM: weighted median method.

We investigated the causal associations between fat distribution indicators and bladder tumors (including benign tumors, BCa, and carcinoma *in situ*). As shown in Figure 2, the results of the IVW method were shown. There was no significant causal association between any of the exposure variables and benign bladder tumors. For carcinoma *in situ*, GFATadj showed a negative association (OR = 0.67, 95% CI = 0.46-0.98, p = 0.038). For BCa, ASATadj (OR = 1.78, 95% CI = 1.27-2.50, p=0.001) and ASAT/GFAT (OR = 1.64, 95% CI = 1.01-2.66, p = 0.047) were positively associated with BCa. We further validated these positive results using MR-Egger and WM methods as complements (Figure 3). For carcinoma *in situ*, the MR-Egger OR direction of GFATadj was opposite to that of IVW; whereas for BCa, the MR-Egger OR directions of ASATadj and ASAT/GFAT were consistent with those of IVWs.

### Effect of circulating metabolites on BCa

**Figure 4:**
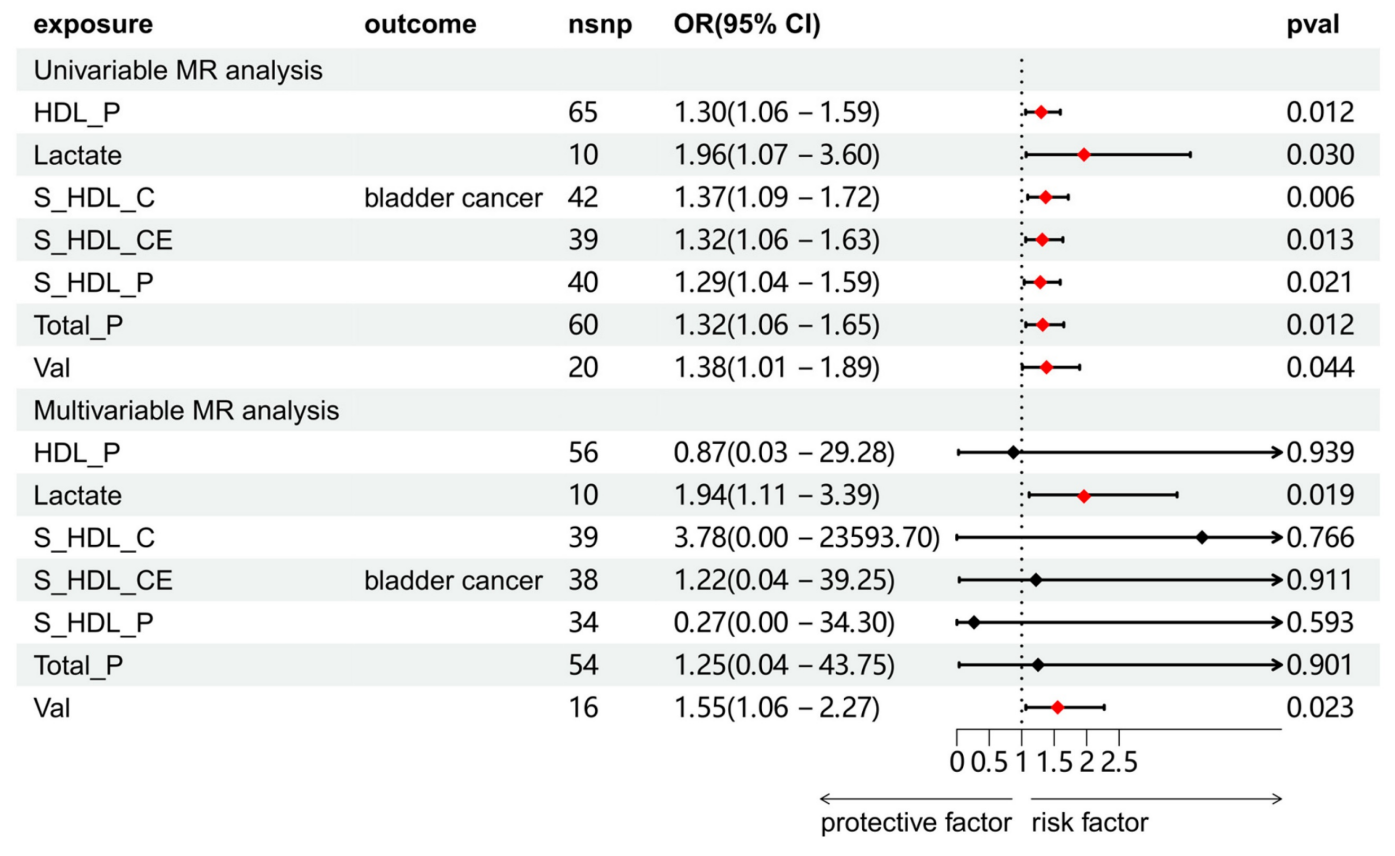
Univariable and multivariable MR analyses of the relationship between circulating metabolites and BCa. Full names of exposure indicators are provided in Table S2.

We explored the association between circulating metabolites and BCa. Among the 168 circulating metabolites, 8 substances showed a significant causal association with BCa, and sensitivity analysis was subsequently conducted on these eight metabolites to eliminate those exhibiting heterogeneity or pleiotropy (Table S6 and S12). A total of 7 substances were screened. Further multivariate MR analysis revealed that lactate and valine were causally associated with BCa (Figure 4).

### Effect of fat distribution on circulating metabolites

The causal relationships between ASATadj, ASAT/GFAT and 2 metabolites (lactate and valine) were further explored. The results showed that only ASAT/GFAT and valine demonstrated a clear causal association. Combined with the results of Figure 4, we established the following causal relationships: ASAT/GFAT to BCa, ASAT/GFAT to valine, and valine to BCa.

### Reverse MR analysis and Steiger directionality test

For the above variables that have a causal association, the reverse MR analysis did not find any potential causal associations among them (Table S14). The Steiger directionality test further validated the causal inference direction from the exposure to the outcome (Table S15).

### Mediation effect of fat distribution on BCa

Based on the established mediating causality, we conducted a mediation analysis using a two-step MR design to explore the mediating effects of valine on BCa. The total effect of ASAT/GFAT on BCa was β0 = 0.492, and the indirect effect mediated by valine was β1*β2 = 0.035, accounting for 7.13% of the ASAT/ GFAT-related BCa risk (95% CI = 3.57%-10.69%, p = 0.045).

### Sensitivity analysis

Although there was heterogeneity in the sensitivity analysis of the causal effect of ASAT/GFAT on valine (p = 0.017), the random-effects IVW model found a causal association between them, and no outliers were identified in the MR-PRESSO analysis, and there was no evidence of horizontal pleiotropy. In addition, the funnel plot and leave-one-out analysis also showed no significant outliers (Figure S2C and S3C). None of the remaining sensitivity analyses showed significant heterogeneity or horizontal pleiotropy (Table 1).

## Discussion

As fat is a microenvironment that promotes tumor growth, fat accumulation has garnered increasing attention. Most previous evidence about fat traits and disease risk comes from retrospective analysis or observational studies, with many using easily available indicators such as BMI, WC, and hip circumference (HC) to replace obesity^15^. However, these indicators are often affected by confounders, and reverse causality can't be excluded. In this study, we utilized publicly available GWAS data and MR analysis to explore the causal relationships between different fat traits and BCa. Our findings suggested that ASATadj and ASAT/GFAT may increase the risk of BCa, with ASAT/GFAT potentially increasing the risk through the mediating effect of valine.

Obesity is an important public health problem worldwide, and is associated with the occurrence and progression of cardiovascular events and cancer, as well as an increase in cancer-related mortality. BMI, WC, HC and waist-to-hip ratio (WHR) are commonly used for obesity assessment due to their ease of measurement; however, their accuracy is inherently limited. For instance, BMI is calculated based on the ratio of weight to height, which has a certain methodological bias. Importantly, it fails to differentiate between visceral fat and subcutaneous fat. While WC can be used to assess the degree of abdominal obesity and predict cancer risk better than BMI, it still suffers from similar limitations and is susceptible to racial differences^16^.

Despite numerous studies, the association between obesity and BCa remains unclear^17^. A large meta-analysis showed that obesity increased the risk of BCa (OR = 1.10, 95% CI = 1.06-1.14)^15^. The positive association between BMI and BCa remained after stratification for smoking status, a well-known risk factor for BCa^18^. Another meta-analysis also confirmed the role of obesity in the incidence of BCa, while height didn't appear to affect the risk of BCa^19^. Interestingly, this study also found that being overweight and having a larger WC were associated with BCa risk in men, but not in women. Conversely, obese women had a greater risk of BCa than men. Another study investigated the effect of BMI on the development of BCa among people with and without abdominal obesity^20^. Individuals with the same BMI may have different fat distribution traits, for example, athletes with a high BMI have more muscle mass than fat. Consistent with previous research, this study confirmed that BMI was indeed a risk factor for BCa, but the association was influenced by abdominal obesity. Among those with a WC less than 90 cm, BMI didn't significantly change the risk of BCa, but among those with a WC greater than 90 cm, there was a significant change in BCa risk as BMI changed. In addition, a recent study that summarized the evidence on the relationship between BMI and a variety of diseases in MR studies^21^. This study provided support for the causal association between obesity and BCa, and also revealed that genetically predicted BMI may increase the risk of BCa (OR = 1.27, 95% CI = 1.05-1.53). However, some cohort studies or case-control studies haven't found a causal relationship between obesity and BCa^22^.

Obesity is primarily associated with the accumulation of body fat. AT, one of the largest endocrine organs in the human body, accounts for approximately 26% and 38% of the body weight in adult men and women, respectively^23^. In adults, adipose tissue mainly consists of white fat, which is categorized into visceral fat and subcutaneous fat^24^. Subcutaneous fat accumulates in areas such as the breast, abdomen, or hips, while visceral fat surrounds vital organs including the gastrointestinal tract and kidneys. Excess fatty acids are initially stored subcutaneously, and once the capacity of subcutaneous fat is reached, additional fat storage occurs in other areas, such as visceral fat^8^. The different distribution locations also determine the local or systemic effects of obesity. Many studies have confirmed the important role of adipose tissue in the occurrence and development of various cancers, such as breast cancer^25^, colorectal cancer^26^, kidney cancer^27^, and prostate cancer^28^. Most of these organs are surrounded by visceral or subcutaneous fat. Our study revealed that ASATadj and ASAT/GFAT may be risk factors for BCa. We hypothesize that this could be attributed to the anatomical location of the bladder. The bladder is located in the pelvic cavity, adjacent to the peritoneum at the top, and is surrounded by soft tissues. Especially its anterior and lateral aspects, which are in direct contact with subcutaneous adipose tissue.

Obesity is considered a chronic subclinical pro-inflammatory environment^29^. Chronic inflammation is associated with several stages of carcinogenesis, including cellular transformation, proliferation, invasion, angiogenesis, metastasis, and more^30^. Both local and systemic inflammation contribute to the onset and progression of BCa. Several signaling pathways have been associated with the onset and progression of BCa during inflammation, including COX-2/nitric oxide synthase (NOS), nuclear factor-kappaB (NF-κB), and PI3K-Akt-mTOR^31^. During inflammation, the activation of inducible nitric oxide synthase (iNOS) leads to the production of nitric oxide (NO), which can impair DNA repair mechanisms and promote angiogenesis^32^. Observational studies indicate that patients with BCa have significantly higher levels of NO compared to the control group^33^. The iNOS is found in BCa patients but not in healthy controls, and inducible NO plays an important role in tumor angiogenesis^34^, ^35^. The NF-κB is considered to be a molecular link between inflammation and cancer^36^. Activation of NF-κB mediates angiogenesis and metastasis in BCa by regulating interleukin-8 (IL-8)^37^. Some cytological studies have shown that adipose-derived stem cells promote the proliferation and invasion ability of BCa cells by secreting proinflammatory cytokines such as IL-6 and IL-8, and play a positive role in tumor progression^38^-^40^. Additionally, the accumulation of adipose tissue is associated with increased levels of oxidative stress. Inflammatory factors and metabolic products secreted by adipose tissue can increase the production of free radicals. These free radicals have carcinogenic effects by inducing DNA damage and mutations, thereby promoting the development of cancer^41^.

As an essential amino acid in the human body, valine is a branched-chain amino acid (BCAA). Felig's study first revealed increased levels of circulating BCAAs in obese individuals^42^. Many studies have also reported a positive association between obesity and circulating BCAA levels^43^-^45^. Our results are consistent with these findings. This phenomenon may be attributed to the inhibition of liver branched-chain α-keto acid dehydrogenase (BCKDH) activity by obesity, which is associated with insulin resistance. This leads to an increase in blood BCAA levels^46^. BCKDH serves as the rate-limiting enzyme in the catabolism of BCAAs, exhibiting the highest enzyme activity in the liver^47^. The metabolism of BCAAs is initiated by BCAA transaminase (BCAT)^47^. Alterations in BCAA metabolism have been observed in various solid tumor environments. For instance, BCAT1, which is highly expressed in glioblastoma, has been found to promote tumor growth^48^. Similarly, high expression of BCAT1 in breast cancer can promote the biogenesis of mitochondria in an mTOR-dependent manner to meet the proliferation needs of tumors^49^. This phenomenon has also been observed in heart and skeletal muscle^50^. In addition, BCAAs also have early diagnostic value for tumors. High levels of circulating BCAAs in the early stage of pancreatic cancer may be associated with atrophy of peripancreatic tissues, which has a predictive ability for diagnosing pancreatic cancer^51^. The urinary valine level in patients with BCa is significantly greater than that in the normal group or patients with a history of BCa^52^, ^53^, and it can also distinguish between high- and low-grade BCa^54^. These studies suggest that BCAAs are involved in the occurrence and development of tumors, which is also supported by our results.

The main strengths of this study lie in its use of MR analysis to investigate the causal relationship between fat distribution and BCa, revealing that accumulation of ASAT increases the risk of BCa. Further univariable, multivariable, and mediation MR analyses suggested that valine may mediate this association. These analyses exhibited no significant heterogeneity or pleiotropy and presented consistent direction and magnitude across methods in IVW, WM, and MR-Egger methods. The causal inference direction from exposure to outcome was validated by reverse MR analysis and Steiger directionality tests.

However, several limitations of this study must be acknowledged. First, the data used in this study primarily come from European populations, and due to the lack of fat distribution data from other ethnic groups, caution is needed when generalizing the results to other populations. Future research should develop and include data from other ethnic groups to further validate the findings. Second, this study primarily infers the causal associations and mediation between fat distribution and BCa based on static genetic variations. In reality, cancer is a result of long-term exposure to risk factors, and fat distribution and blood metabolites can be influenced by time and lifestyle factors. Future research should employ longitudinal data to explore these dynamic effects. Third, BCa development is a multifactorial and multistep process. The pathway from fat distribution to BCa involves multiple mechanisms, and our study represents only one of these pathways. Further research is needed to elucidate the complex associations between these factors. Additionally, BCa risk is influenced by various factors, and genetic variations alone may not provide a comprehensive risk prediction. The findings of this study should be considered as part of a broader risk assessment. Future research should focus on integrating genetic variations with other risk factors to improve risk prediction models.

Many studies have confirmed the role of visceral fat in the risk of cardiovascular diseases and various cancers. Indeed, reducing visceral fat accumulation is crucial for lowering the risk of these conditions. However, fat tissue tends to be preferentially stored subcutaneously, and the impact of ASAT on health is also worth noting. Our research found that accumulation of ASAT increases the risk of BCa, and the blood metabolite valine may be involved in this process. Therefore, reducing ASAT accumulation is important for alleviating the disease burden of BCa. With advancements in measurement technologies, monitoring ASAT will become simpler and more efficient. Our findings provide important insights for BCa prevention strategies.

## Conclusion

In summary, our findings suggest that ASAT may play a significant role in the onset and progression of BCa, with valine potentially involved in this process. Maintaining a healthy weight and reducing the accumulation of subcutaneous fat can help reduce the disease burden of BCa.

## Supplementary Material

Supplementary figures and tables.

## Figures and Tables

**Figure 5:**
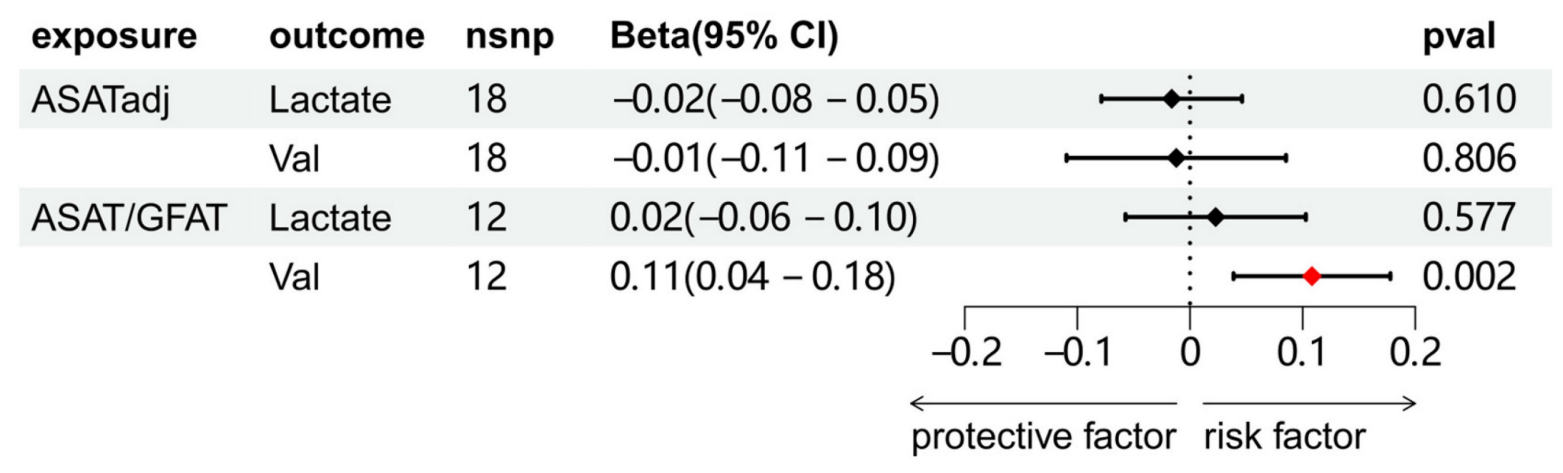
Two-sample MR analysis results of the causal relationship between ASATadj, ASAT/GFAT and 2 metabolites.

**Figure 6:**
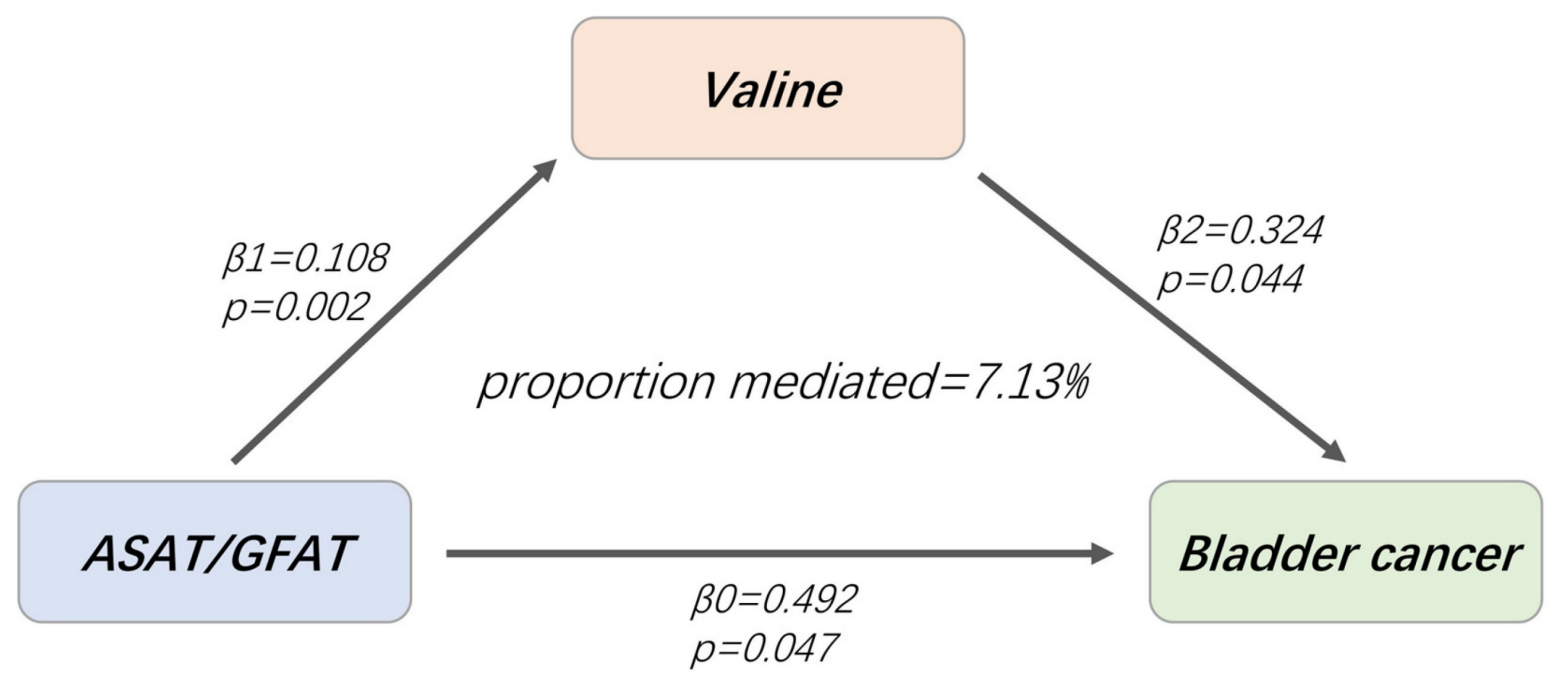
Valine mediates the causal effect of ASAT/GFAT on BCa. β0: the total effect of ASAT/GFAT on BCa; β1: the causal effect of ASAT/GFAT on valine; β2: the causal effect of valine on BCa.

**Table 1:** Heterogeneity test and horizontal pleiotropy test

Exposure	Outcome	Heterogeneity			MR-Egger pleiotropy	
		Cochrane's Q	p		Intercept	p
ASATadj	BCa	11.775	0.759		0.042	0.383
ASAT/GFAT	BCa	13.657	0.189		0.013	0.811
Valine	BCa	18.341	0.500		0.003	0.874
ASAT/GFAT	Valine	23.107	0.017		-0.006	0.430

## Abbreviations

BCa: bladder cancer
BMI: body mass index
WC: waist circumference
MR: mendelian randomization
GWAS: genome-wide association studies
SNP: single nucleotide polymorphism
VAT: visceral adipose tissue
SAT: subcutaneous adipose tissue
ASAT: abdominal subcutaneous adipose tissue
GFAT: gluteofemoral adipose tissue
VATadj: VAT adjusted for BMI and height
ASATadj: ASAT adjusted for BMI and height
GFATadj: GFAT adjusted for BMI and height
IV: instrumental variable
IVW: inverse variance weighted
WM: weighted median
MR-PRESSO: mendelian randomization pleiotropy residual sum and outlier
OR: odds ratio
95% CI: 95% confidence interval
BCAA: branched-chain amino acid
BCKDH: branched-chain α-keto acid dehydrogenase
BCAT: branched-chain amino acid transaminase
iNOS: inducible nitric oxide synthase
NF-κB: nuclear factor-kappaB
IL: interleukin
