# Supplementary material for: Causal Association of Adipose Tissue with Bladder Cancer and the Mediating Effects of Circulating Metabolites: A Mendelian Randomization Study
Source: J Cancer. 2024 Oct 21;15(20):6521–30. doi: 10.7150/jca.100152 (PMC11632975; doi:10.7150/jca.100152)
Supplement: Supplementary file 1 — Supplementary figures and tables. [file jcav15p6521s1.zip › Supplementary figures.pdf]

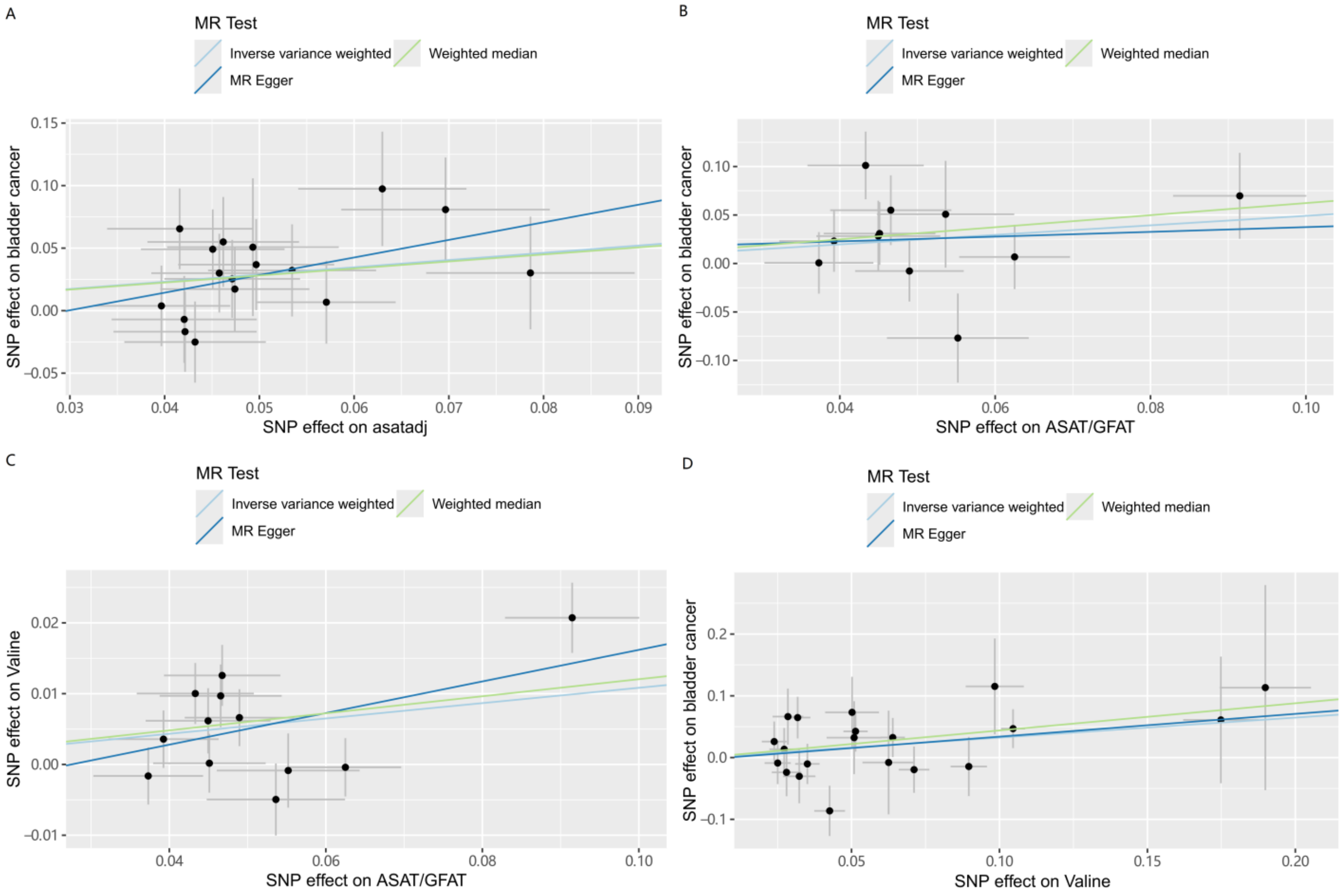

Figure S1: Scatter plots of the main causality between exposure and outcome. (A) ASATadj and bladder cancer; (B) ASAT/GFAT and bladder cancer; (C) ASAT/GFAT and valine; (D) valine and bladder cancer.

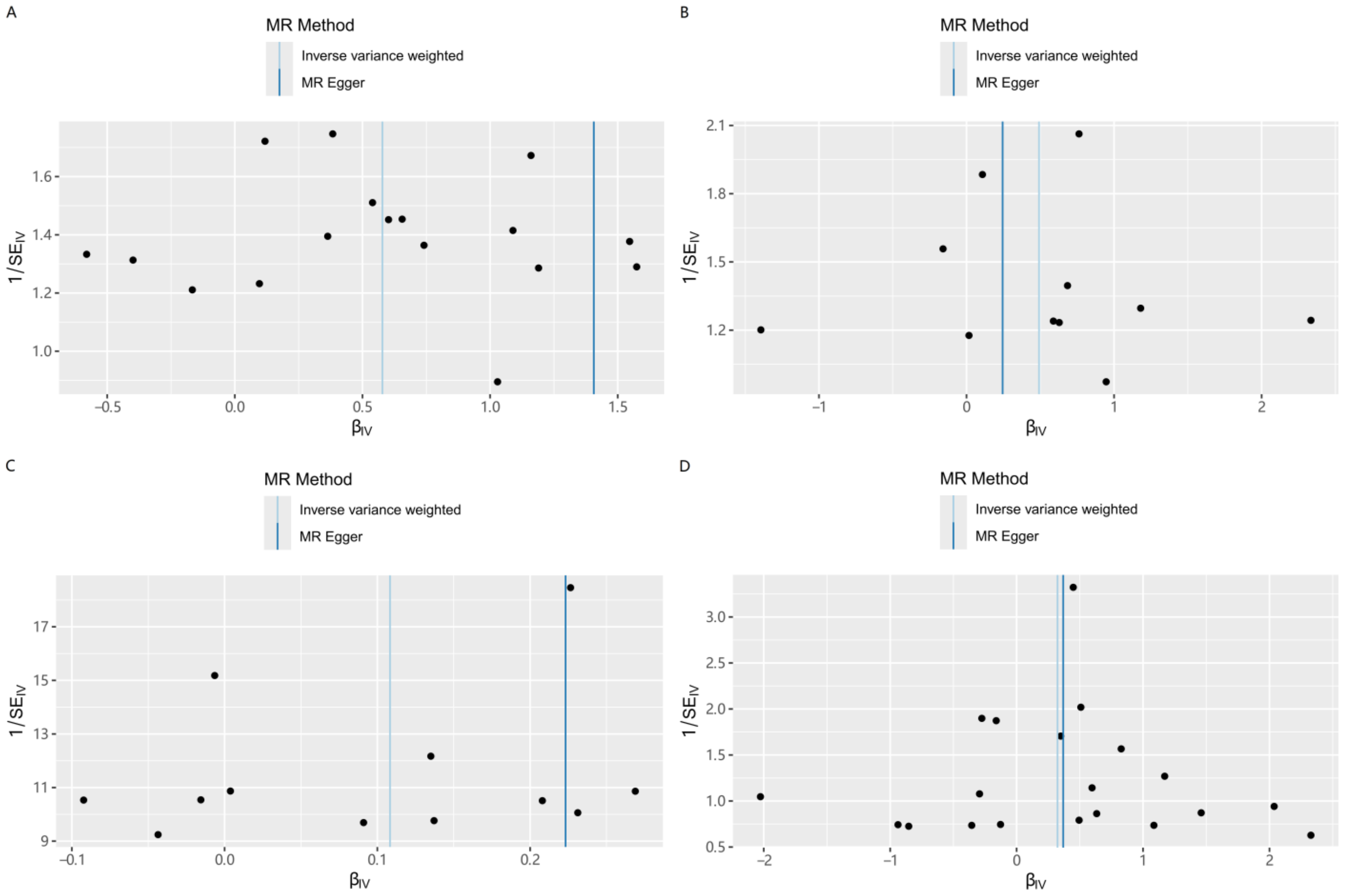

Figure S2: Funnel plots of the main causality between exposure and outcome. (A) ASATadj and bladder cancer; (B) ASAT/GFAT and bladder cancer; (C) ASAT/GFAT and valine; (D) valine and bladder cancer.

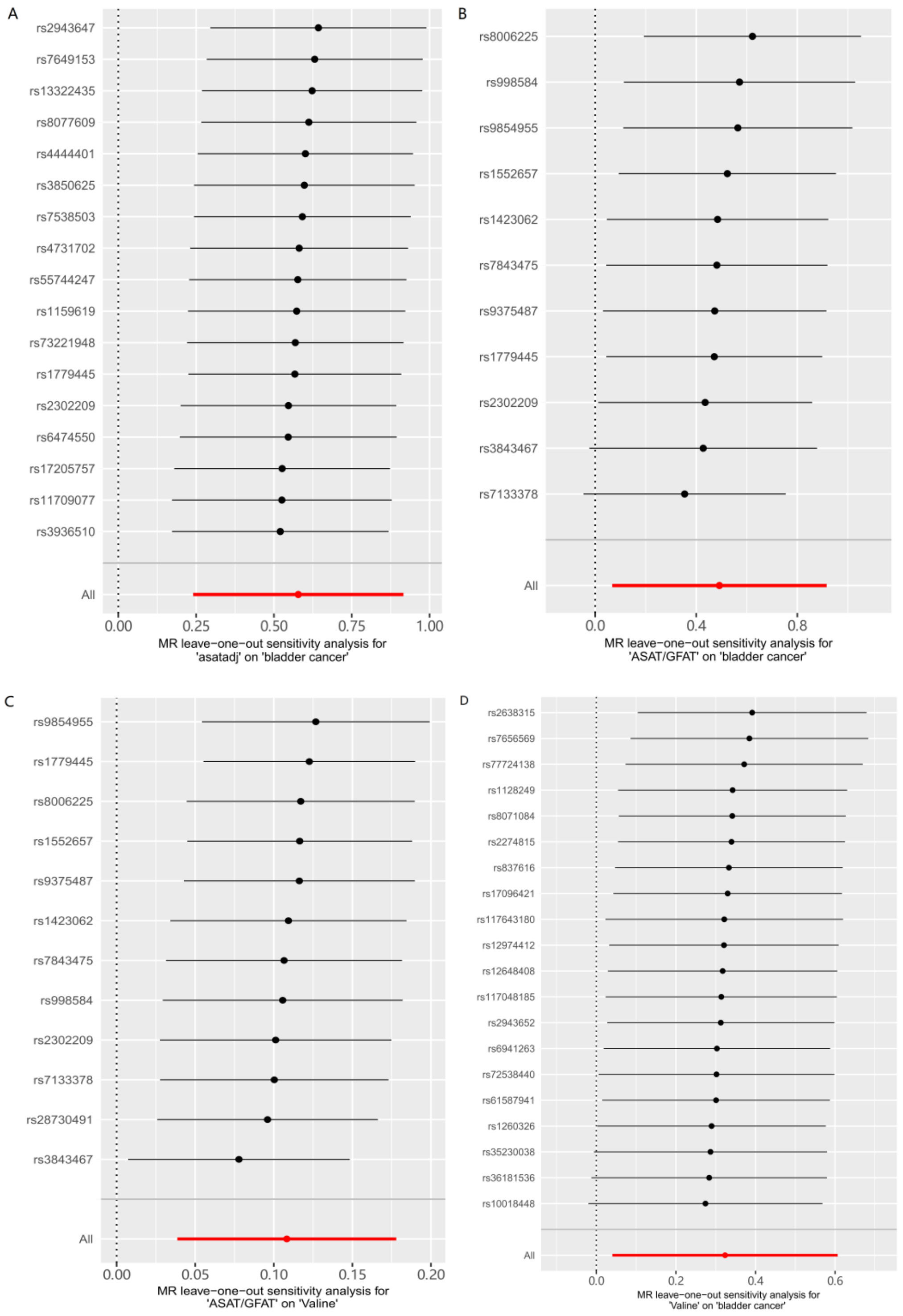

Figure S3: Leave-one-out analysis for the impact of individual SNPs on the main causality between exposure and outcome. (A) ASATadj and bladder cancer; (B) ASAT/GFAT and bladder cancer; (C) ASAT/GFAT and valine; (D) valine and bladder cancer.
